# Real-time measurement of adenosine and ATP release in the central nervous system

**DOI:** 10.1007/s11302-020-09733-y

**Published:** 2020-10-06

**Authors:** Nicholas Dale

**Affiliations:** School of Life Sciences, University of Warwick, Coventry, CV4 7AL UK

**Keywords:** Biosensor, Ischaemia, Motor pattern generation, Chemosensory, Breathing, Connexin

## Abstract

This brief review recounts how, stimulated by the work of Geoff Burnstock, I developed biosensors that allowed direct real-time measurement of ATP and adenosine during neural function. The initial impetus to create an adenosine biosensor came from trying to understand how ATP and adenosine-modulated motor pattern generation in the frog embryo spinal cord. Early biosensor measurements demonstrated slow accumulation of adenosine during motor activity. Subsequent application of these biosensors characterized real-time release of adenosine in in vitro models of brain ischaemia, and this line of work has recently led to clinical measurements of whole blood purine levels in patients undergoing carotid artery surgery or stroke. In parallel, the wish to understand the role of ATP signalling in the chemosensory regulation of breathing stimulated the development of ATP biosensors. This revealed that release of ATP from the chemosensory areas of the medulla oblongata preceded adaptive changes in breathing, triggered adaptive changes in breathing via activation of P2 receptors, and ultimately led to the discovery of connexin26 as a channel that mediates CO_2_-gated release of ATP from cells.

## Introduction

I vividly remember the first time I heard Geoff Burnstock give a seminar. It was in 1994, and although I had known Geoff for quite some time, I had never heard him deliver a lecture. So, when he came to Bristol to give a major talk, I went with great eagerness, but also not knowing quite what to expect. What unfolded over the course of about an hour was one of the most inspiring lectures I had ever heard. At the time I had only a dim, verging on nonexistent, grasp of purinergic signalling. Geoff, at the height of his powers, convinced me, and I suspect everyone else in the audience that the purines were pretty much the answer to every physiological problem out there. I was so inspired that I got into my lab the very next day and started dabbling with ATP and adenosine. I had been running my own independent lab for about 5 years. I was working on the neuronal mechanisms of spinal motor pattern generation in the frog embryo, trying to build Hodgkin-Huxley inspired models of the circuit, so it was natural to apply ATP and adenosine to the spinal cord and look at the consequences for motor pattern generation. Immediately I realized that there was something very interesting: both ATP and adenosine had powerful but opposing actions, on the neural circuitry that generated the swimming motor pattern. Two years of hard work, and self-tuition in the arts of purinergic signalling, resulted in a paper that described an excitatory action of ATP (mediated via an effect of voltage gated K^+^ channels) and an inhibitory action of adenosine (through inhibition of voltage gated Ca^2+^ channels via A1 receptors) [1]. It was clear that the inhibitory action of adenosine had something to do with controlling the spontaneous slowing of the swimming motor pattern and its eventual termination. Courtesy of Geoff’s great lecture, and subsequent reading of the literature, I was also aware that the adenosine might arise from conversion of ATP that had been released into the extracellular space [2]. This led to a puzzle: if the released ATP, which was excitatory, was converted to adenosine, which was inhibitory, how did this give rise to the observed temporal control of motor pattern generation? One way that this could arise would be if the production of adenosine was delayed with respect to the release of ATP. In fact, there was already a mechanism in the literature whereby ATP and ADP could inhibit the ecto-5′-nucleotidase that converts AMP to adenosine that could introduce such a hypothesized delay [3–5]. It was these thoughts that led me to the realization that I needed to test these ideas by measuring the release of adenosine in real time. After further scanning of the literature, I realized that the only way to achieve this was to invent a biosensor for adenosine. What follows is a not a scholarly review of purine measurements, but a personal account of some of the science, in which I have been involved, which flowed from the inspiration that Geoff’s talk gave me.

## Real-time measurement of adenosine

**Fig. 1:**
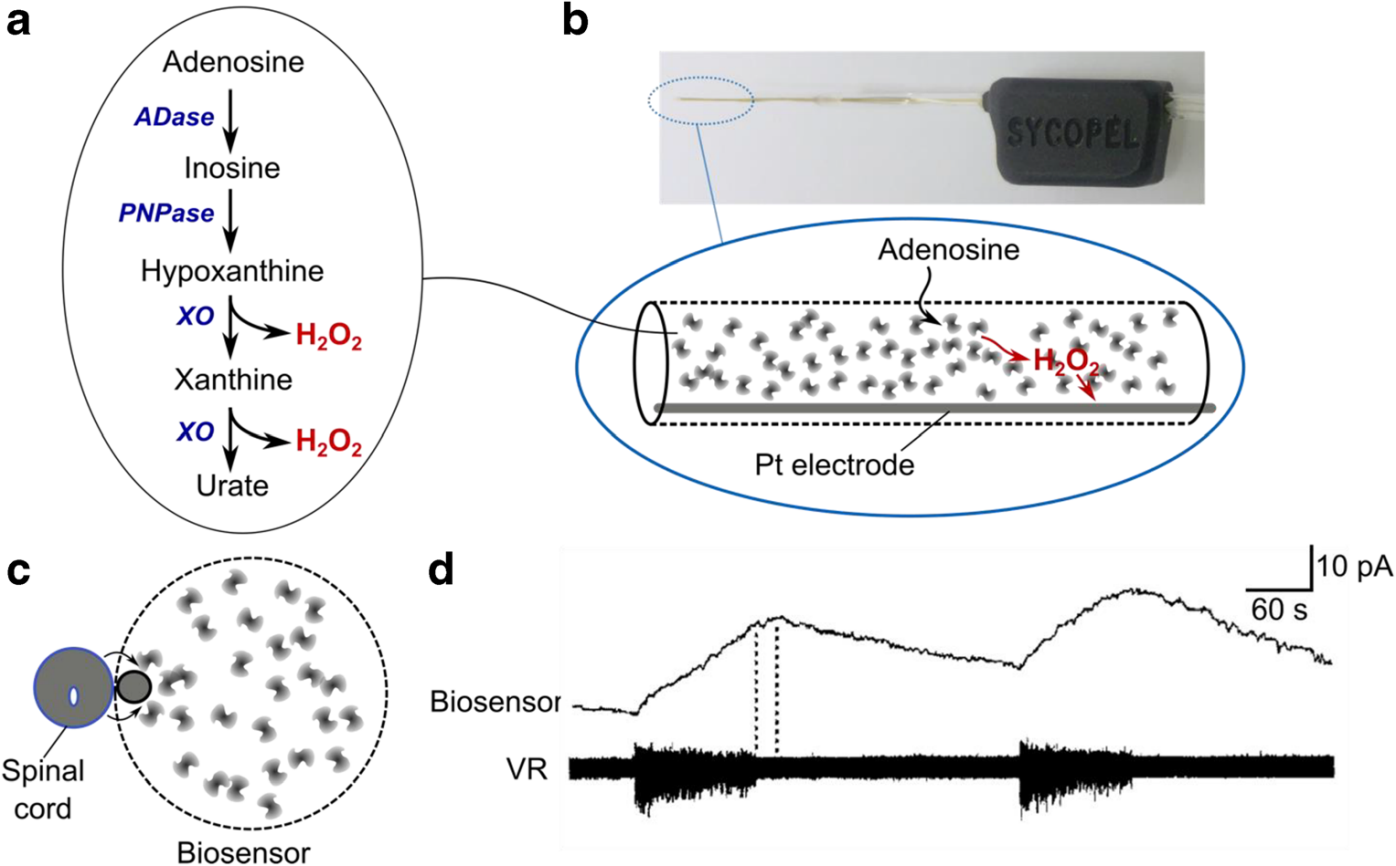
First real-time recording of adenosine during neural activity. (**a**) Enzymatic cascade for the adenosine biosensor. ADase, adenosine deaminase; PNPase, purine nucleoside phosphorylase; XO, xanthine oxidase. (**b**) Image of the microdialysis electrode assembly use for the original adenosine biosensor with schematic showing enzymes in solution, supporting the production of H_2_O_2_ in proportion to the concentration of adenosine (able to diffuse through the porous wall of the electrode) which was detected by the Pt electrode held at 650 mV with respect to an Ag/AgCl reference. (**c**) Schematic of arrangement for recording adenosine release during fictive swimming in the tadpole. The biosensor and spinal cord are shown in cross section to show their relative sizes. Diffusion of adenosine from the spinal cord to the biosensor is indicated by arrows; note that the position of the Pt electrode inside the microdialysis tube was crucial to enable reliable detection. (**d**) Recording of the real-time release of adenosine during swimming acting indicated by motor nerve activity on the ventral root (VR). Trace reproduced from reference [6]

To make a biosensor for adenosine, I borrowed from nature the enzymes that are involved in processing purines: adenosine deaminase, purine nucleoside phosphorylase, and xanthine oxidase [6]. This cascade will successively convert adenosine via inosine, hypoxanthine, and xanthine to uric acid. Xanthine oxidase produces H_2_O_2_, which is easy to detect electrochemically (Fig. 1a). Of course, a biosensor that involves 3 enzymes will be sensitive to substrates for all 3 enzymes, so to test whether it specifically detected adenosine, I utilized an inhibitor of adenosine deaminase, coformycin. To make the biosensor, I loaded these three enzymes in aqueous solution into a very fragile microdialysis electrode assembly (Fig. 1b). The idea was that adenosine, released from tissue, would diffuse through the dialysis membrane, be metabolized by the enzymes within the electrode, and thus generate the H_2_O_2_ for electrochemical detection via the internal electrodes (Fig. 1b, c). This assembly, at 250 μm in diameter, was bigger than the spinal cord I was trying to measure from (diameter about 70 μm), and at first, I saw nothing. But one day, after numerous attempts, I detected an adenosine signal. By chance it turned out that the assembly I had used that day had the Pt wire for detecting the H_2_O_2_ in a position that led to it being right next to the spinal cord (Fig. 1c). Reliable detection of adenosine was accomplished once I had convinced the manufacturer of the microdialysis assembly to make them all with that particular arrangement of the internal electrodes (Fig. 1d). My recordings showed that adenosine was produced gradually during motor activity and gave experimental support for the hypothesis of delayed production in the extracellular space [6, 7].

I realized that the adenosine biosensor was potentially quite useful and thought that finding a few collaborators to pursue other questions would be interesting. So, once again, I turned to Geoff and asked him who he thought might be worth contacting. Geoff suggested that his colleague, Mike Spyer, a physiologist at UCL with wide interests in autonomic control who at the time was in the Royal Free Medical School and had an office close to Geoff’s, would be good to talk to. This was an inspired recommendation, as Mike Spyer had seen my adenosine biosensor paper and had already written to me. Courtesy of a recommendation from Charles Kennedy, I also met up with Bruno Frenguelli, then at Dundee, to discuss collaborations. My collaborations with Mike Spyer and Bruno Frenguelli turned out to be long lasting, fun, very productive, and instrumental in providing the scientific stimulus to develop further the biosensor technology for adenosine and ultimately a biosensor for ATP and other neuroactive compounds.

## Measurement of adenosine release: in vitro stroke models to clinical studies

**Fig. 2:**
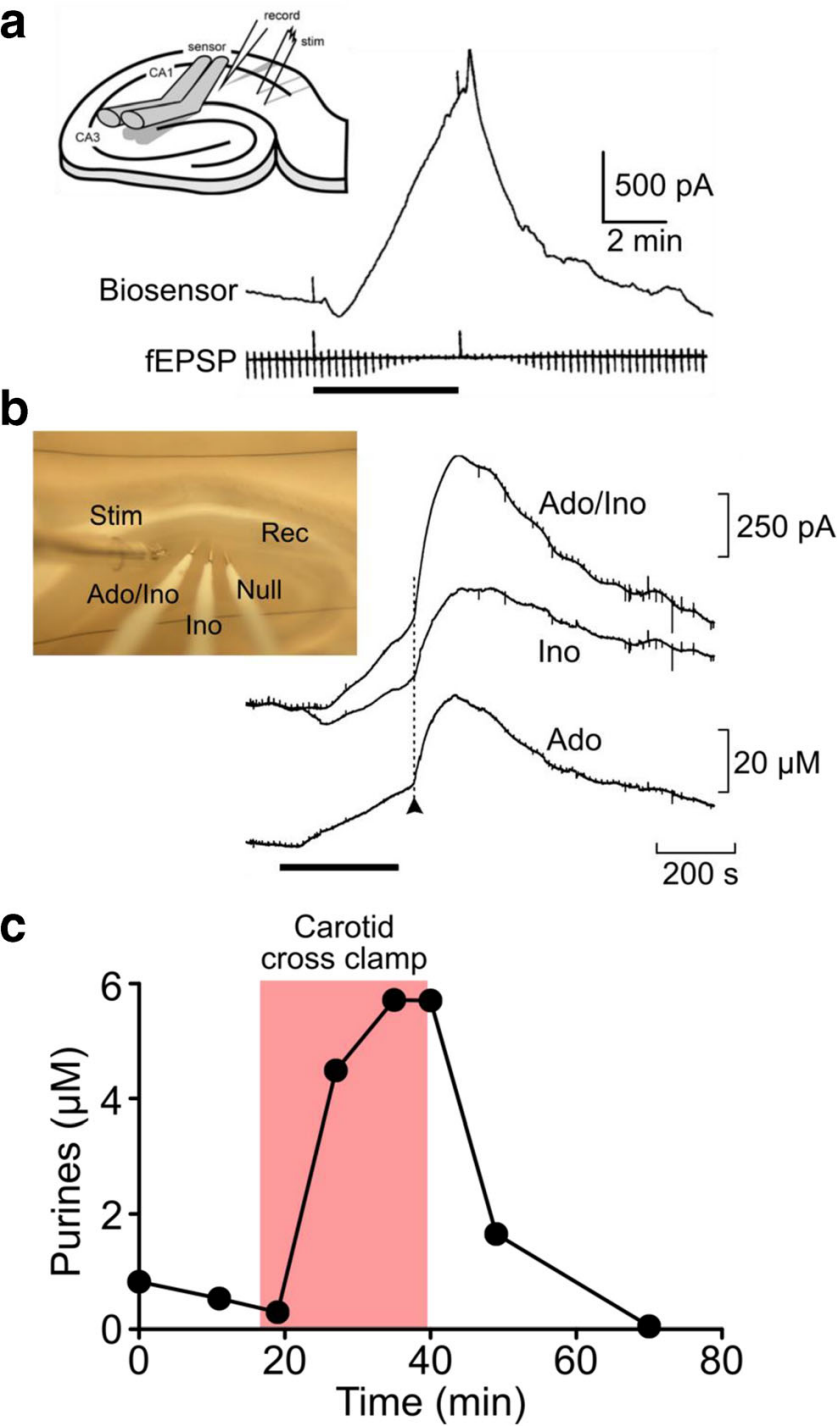
Measurement of adenosine release during hypoxia/ischaemia. (**a**) Real-time recording of adenosine release from a hippocampal brain slice during a hypoxic episode (black bar). Synaptic transmission was recorded simultaneously (fEPSP). The inset shows the recording arrangement; this was made with the same type of microdialysis electrode as show in Fig. 1, except that it had two barrels which could be loaded with different enzyme combinations. Reproduced from [17]. (**b**) Microelectrode biosensor recordings of adenosine release during hypoxia (black bar). The inset shows a picture of the biosensors inserted into a hippocampal slice (the shiny metallic portion within the slice is the sensing tip). Measurement of purine release within the slice showed new features such as the post-hypoxic purine efflux (occurring at arrow head). Differential measurements between a biosensor with all three enzymes (Ado/Ino) and one only having PNPase and XO (Ino) allowed specific recording of the adenosine signal (Ado). Reproduced from [18]. (**c**) Measurement of purines (combination of adenosine, inosine, hypoxanthine) in arterial blood of a patient undergoing carotid endarterectomy. Note that purines are rapidly released from the brain when the carotid artery is cross clamped (pink box). Reproduced from [19]

At the time that Bruno Frenguelli and I started to collaborate in early 1998, Bruno’s interest was in how the brain dealt with metabolic stress. Bruno had developed an in vitro model of a stroke in a dish, whereby a hippocampal brain slice could be starved of O_2_ (hypoxia) or in a more severe model deprived simultaneously of O_2_ and glucose (“ischaemia”). Recording of synaptic transmission within the slice (the hippocampus with its well-understood neuronal architecture and synaptic connectivity being very advantageous for this) allowed a physiological read-out of the effect of hypoxia/ischaemia. It was known from work of many investigators that, during hypoxia/ischaemia, adenosine was released from neural tissue [8–16]. This work mainly used microdialysis or related techniques to collect perfusate and then analyze this post hoc via HPLC—a laborious and time-consuming enterprise. Bruno and others had pharmacological evidence in vitro that the depression of synaptic transmission during hypoxia/ischaemia was mediated via adenosine A1 receptors. Thus, when we started to work together, we had a clear expectation that we should see adenosine release during the hypoxic/ischemic episodes. The advantage of using a biosensor was that we could not only detect the release of adenosine but also examine its effect on synaptic transmission in real time. Our recordings allowed us to determine the temporal relationship between adenosine and its action on synaptic transmission (Fig. 2a), to show that adenosine release did not require extracellular Ca^2+^ and that the amount of adenosine depleted with successive hypoxic/ischaemic episodes [17, 20].

Nevertheless, although we had the best, and at the time only, real-time method of measuring adenosine release, our work together made us realize the limitations of what we had. The biosensors were big and bulky, very fragile, and difficult to use. The size and fragility meant that we were unable to put the sensors into the tissue (something referees of our papers were very keen for us to do)—instead we had to lay them on top. It was these limitations that drove development of better, smaller, more sensitive, and faster responding biosensors. The first-generation adenosine biosensor used a microdialysis assembly with the enzymes being present in aqueous solution. I reasoned that if we stuck the enzymes directly to the electrode (by some as yet unknown method), we would have a much better, smaller, and more robust device.

After exploring the biosensor literature, I decided to follow the work of Serge Cosnier [21] and utilize the pyrrole derivatives he had described to make a microelectrode biosensor for adenosine. This required organic synthesis and led to Enrique Llaudet joining the team to make the pyrrole derivatives and fabricate the biosensors [22]. We used these methods for about 3 years but eventually replaced them with a silicate sol gel methodology that we invented and which gave more reliable and sensitive biosensors [23]. In fact, it was our development of the sol gel techniques that made it viable to set up a company, Sarissa Biomedical Ltd., to make and sell biosensors to the scientific community, as by now I had far more people wishing to use these biosensors than I could possibly collaborate with.

The microelectrode biosensors allowed us to place the biosensors inside the tissue. This gave important new insights into the production of purines during stroke-like conditions. We were able to demonstrate that in this case (unlike the tadpole spinal cord), adenosine was released directly and did not arise from prior release of ATP [24]. We refined our understanding of the temporal relationship between adenosine production and its effect on synaptic transmission and found that not only was there progressive release of adenosine during the ischemic episode but that reoxygenation of the tissue greatly accelerated purine release [18, 24] (Fig. 2b). This had not been well described before, but might contribute to the phenomenon of reactive hyperaemia [25] or indeed reperfusion injury [26], as the enhanced purine release would give rise to increased free radical production via H_2_O_2_ from the actions of endogenous xanthine oxidase.

By now we realized that the release of purines during in vitro stroke models might be paralleled by similar observations in the human brain during actual strokes and that adenosine and the purines might serve as sensitive indicators of human cerebral ischemia. However, the biosensors that we used for the physiological experiments were not sufficiently selective for measuring purines in blood, meaning that they were not suitable for any clinical studies. To address this issue, Faming Tian and I developed a next-generation biosensor that had an electrochemical mediator to provide excellent selectivity against electroactive interferences in blood opening the way for clinical applications [27]. Sarissa Biomedical has proven crucial in developing the technology further and thus providing a way to translate our advances in purine biosensing into practical applications. In 2017, via a collaboration with Chris Imray, a vascular surgeon at the University Hospitals of Coventry and Warwickshire, we showed that purines are released from the brain and detectable in the arterial blood of patients undergoing elective carotid artery surgery (and hence subjected to temporary unilateral cerebral hypoxia/ischaemia) [19] (Fig. 2c). A first trial of a very early prototype purine biosensing device “SMARTCap” showed, in a reasonably large cohort, that the purines of stroke patients are elevated compared with healthy controls and patients with mimicking nonstroke conditions [28]. Further trials of the latest prototype device “SMARTChip” are continuing to test the diagnostic accuracy of purines as an aid to detecting stroke.

## Microelectrode biosensors for ATP: chemosensory control of breathing

**Fig. 3:**
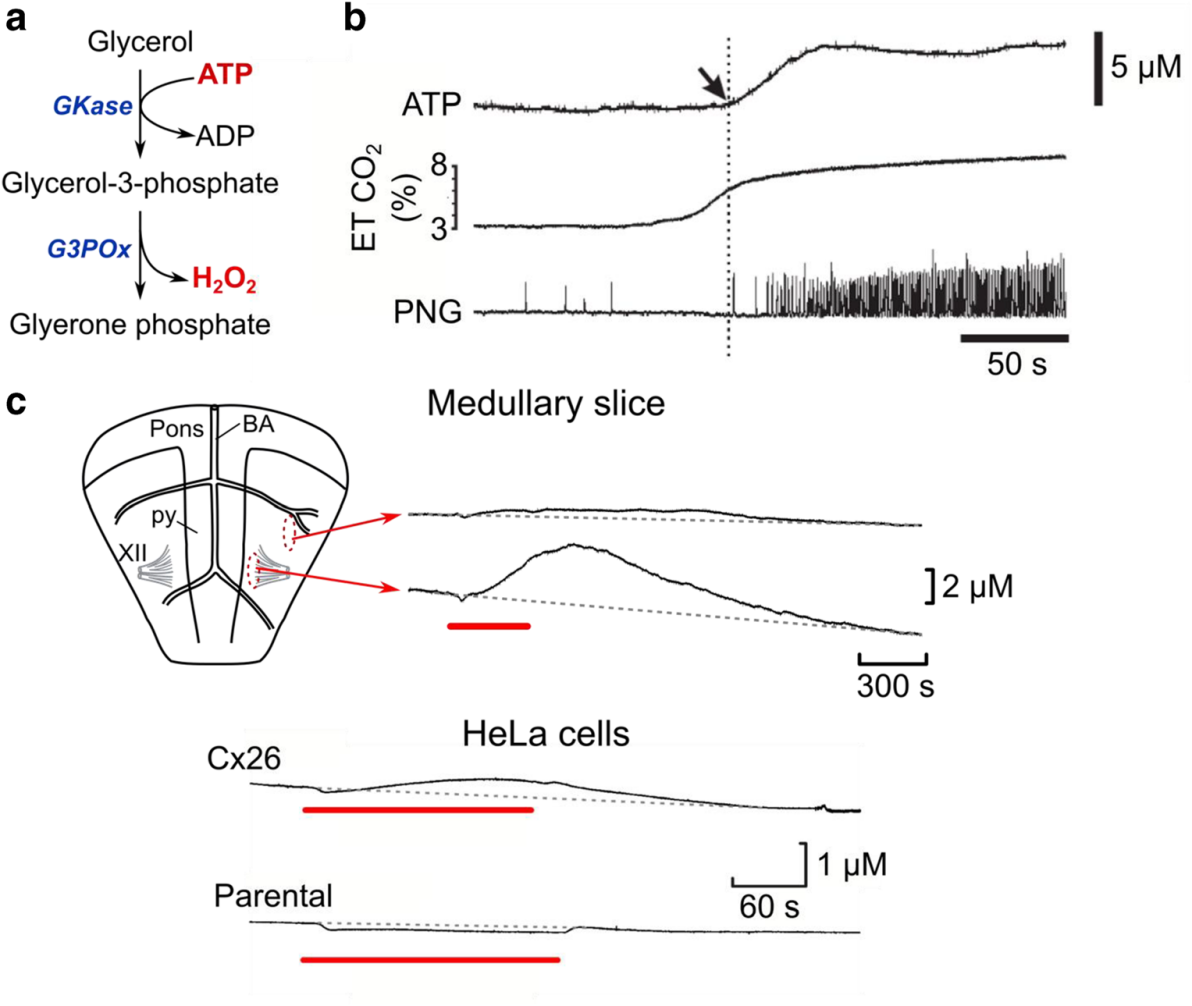
Real-time measurement of ATP release with ATP microelectrode biosensors. (**a**) Enzymatic cascade for the detection of ATP. GKase, glycerol kinase; G3Pox, glycerol-3-phosphate oxidase. This cascade is sensitive only to ATP if glycerol concentrations are at least 0.5 mM. (**b**) Measurement of ATP release during hypercapnia from the ventral surface of the medulla oblongata of anaesthetized artificially ventilated rat. Note that ATP release occurs before the enhancement of breathing as indicated by the phrenic nerve recording (PNG, arrow on ATP biosensor trace and dotted line). End tidal (ET) CO_2_ is also shown. Modified from [31]. (**c**) In vitro models of hypercapnic ATP release. Isolated ventral medullary slice showing ATP release from a caudal region located next to the pyramids (py) and XII^th^ nerve (XII), but not from a much more lateral position. BA -basilar artery. Modified from [32]. CO_2_-dependent ATP release can be recapitulated in HeLa cells that express connexin26 (Cx26) but nor parental HeLa cells that do not express this connexin (modified from [33]). Red bars indicate period of hypercapnia (60 mmHg PCO_2_)

My continued collaboration with Mike Spyer provided a spur to develop a biosensor for ATP. As Mike had obtained evidence that linked ATP signalling to the chemosensory control of breathing [29, 30], the ability to measure ATP release from the chemosensory nuclei in the medulla oblongata was an urgent priority. In developing an ATP biosensor, I had determined that the enzymatic cascade glycerol kinase and glycerol-3-phosphate oxidase would be advantageous (Fig. 3a) [34]. This cascade is sensitive to ATP, if glycerol is provided in the medium, and over the course of many studies, we have found that a concentration of glycerol sufficient to saturate the enzyme is well tolerated by tissue. However, we found it very difficult to find conditions that allowed entrapment of these enzymes on a microelectrode while retaining sufficient sensitivity to make a useful ATP biosensor. We eventually solved this problem with our new sol gel deposition method [35], and returned to work with Mike Spyer, who had now been joined by Alex Gourine, on the problem of CO_2_ chemosensitivity and the control of breathing.

Very quickly, we found that during a hypercapnic episode, ATP was released from proscribed areas of the ventral medullary surface that corresponded to the regions that had been linked to pH/CO_2_ chemosensitivity [31]. Crucially the ATP was released before any adaptive change in breathing, suggesting a causal role between ATP release and the increase in minute ventilation (Fig. 3b). This causal relation was confirmed through use of ATP receptor antagonists [31]. Subsequent work on this problem by Alex Gourine has highlighted an important role of astrocytes in the detection of pH and subsequent release of ATP [36, 37]. In my lab, we looked further at the mechanisms of ATP release and identified that this occurred through hemichannels of the gap junction protein connexin26 (Cx26) (Fig. 3c). We were able to link Cx26 to the chemosensory control of breathing with pharmacological tools [32]. However, we found that Cx26 seemed to respond directly to CO_2_ rather than pH [32, 33]. Subsequent mutational analysis revealed the key residues involved and a potential mechanism that involved a proposed carbamylation of a specific lysine residue in Cx26 by CO_2_ [38]. Cx26 is thus a CO_2_-gated channel capable of releasing ATP. We have recently used our structural understanding of how CO_2_ interacts with Cx26 to develop a dominant negative construct that coassembles with endogenous wild-type Cx26 to remove CO_2_ sensitivity from the resulting heteromeric channel [39]. This genetic approach has allowed us to demonstrate that direct CO_2_ detection via Cx26 contributes about half of the adaptive response to hypercapnia generated by the central chemosensors. There is a population of specialized glial cells present in a circumscribed nucleus—the caudal parapyramidal area that directly detect CO_2_ via Cx26 and regulate breathing via the release of ATP [39]. Very recently, we have taken this down to the atomic level by solving cryoEM structures for Cx26 at different levels of PCO_2_ to directly demonstrate the carbamylation mechanism and understand the conformational changes trigged by CO_2_-binding that lead to channel gating [40].

In giving this personal account, I have tried to show that, like all great scientists, Geoff’s work had far reaching effects on the science of others. For me, it is how Geoff’s inspirational lecture some 26 years ago, and his larger than life effect on the field of purinergic signalling, inspired me to follow an interlinked thread of scientific exploration. At one end of the scientific spectrum, this initial spark led to development of biosensors to analyze purine release and now extends to their application in the clinical diagnosis of stroke, and at the other end of the spectrum, a sub-2 Å structure of the protein that acts as a receptor for CO_2_ can release ATP in response to fluctuations of PCO_2_ and plays a key role in vertebrate physiology.
